# In Vitro Characterization of Sphingosine 1-Phosphate Receptor 1 (S1P_1_) Expression and Mediated Migration of Primary Human T and B Cells in the Context of Cenerimod, a Novel, Selective S1P_1_ Receptor Modulator

**DOI:** 10.3390/ijms23031191

**Published:** 2022-01-21

**Authors:** Lisa Schlicher, Paulina Kulig, Audrey von Münchow, Mark J. Murphy, Marcel P. Keller

**Affiliations:** 1 Idorsia Pharmaceuticals Ltd., 4123 Allschwil, Switzerland; paulina.kulig@idorsia.com (P.K.); lilly.von.muenchow@bucher.ch (A.v.M.); mark.murphy@idorsia.com (M.J.M.); 2 Bucher Biotec AG, 4051 Basel, Switzerland

**Keywords:** S1P_1_ receptor modulator, lymphocyte, chemotaxis, glucocorticoids, systemic lupus erythematosus (SLE)

## Abstract

Cenerimod is a potent, selective sphingosine 1-phosphate receptor 1 (S1P_1_) modulator currently investigated in a Phase IIb study in patients with systemic lupus erythematosus (SLE) (NCT03742037). S1P_1_ receptor modulators sequester circulating lymphocytes within lymph nodes, thereby reducing pathogenic autoimmune cells (including T and B lymphocytes) in the bloodstream and inflamed tissues, making them an effective therapeutic concept for autoimmune disorders. Although the effect of S1P receptor modulators in reducing circulating lymphocytes is well documented, the precise molecular role of the S1P_1_ receptor on these cell types is not fully understood. In this study, the mode of action of cenerimod on human primary lymphocytes in different activation states was investigated focusing on their chemotactic behavior towards S1P in real-time, concomitant to S1P_1_ receptor expression and internalization dynamics. Here, we show that cenerimod effectively prevents T and B cell migration in a concentration-dependent manner. Interestingly, while T cell activation led to strong S1P_1_ re-expression and enhanced migration; in B cells, an enhanced migration capacity and S1P_1_ receptor surface expression was observed in an unstimulated state. Importantly, concomitant treatment with glucocorticoids (GCs), a frequently used treatment for autoimmune disorders, had no impact on the inhibitory activity of cenerimod on lymphocytes.

## 1. Introduction

Sphingosine 1-phosphate (S1P) is a bioactive sphingolipid that acts as a ligand on a group of five G-protein-coupled receptors S1P_1_–S1P_5_. Each receptor subtype shows a distinct expression profile, spanning a wide range of different cell types and tissues, mainly associated with functions in the central nervous system, the vascular system and immune system [1]. Specifically, the S1P_1_ receptor plays a key role in the functioning of endothelial cells and lymphocytes. In the case of lymphocytes, the S1P_1_ receptor is crucial in orchestrating the egress of T and B cells from secondary lymphoid organs to the lymphatic system and blood by receptor-mediated chemotaxis towards an S1P concentration gradient which is highest in blood/lymph. Due to the high concentrations of S1P in blood and lymph, a rapid internalization and thereby desensitization of the S1P_1_ receptor occurs on lymphocytes present there. Within lymphoid organs and tissue, the presence of a degradative S1P-lyase maintains low S1P levels, allowing the re-expression of the receptor—the required precondition for subsequent lymphocyte egress, thereby closing the cycle of lymphocyte trafficking [2]. 

Due to its fundamental role in immune cell trafficking, S1P_1_ receptor-mediated chemotaxis has been implicated in a variety of immune-related diseases and has therefore become an attractive therapeutic target. Fingolimod (FTY720), a non-selective S1P receptor (S1PR) modulator, had been approved by the FDA for the treatment of relapsing multiple sclerosis (RMS) as the first drug with this mode of action. Subsequently, various other selective S1PR modulators followed in the area of MS (ozanimod, ponesimod and siponimod) and ulcerative colitis (UC; ozanimod). These modulators act as functional antagonists on the S1P_1_ receptor by leading to sustained receptor internalization and desensitization. As a result, lymphocytes lose their ability to “sense” the S1P gradient, preventing their egress from the secondary lymphoid organs and leading to a decrease in lymphocyte counts, including pathogenic autoimmune cells in the bloodstream and in inflamed tissues [1,3,4,5].

Systemic lupus erythematosus (SLE) is another indication for which this mode of action may be an appropriate means of intervention. This autoimmune disease with a high unmet medical need can affect virtually any organ of the body ranging from mild to life-threatening manifestations including irreversible organ(s) dysfunction and reduced life span. The incomplete understanding of SLE pathogenesis makes drug development in this disease particularly challenging. However, it has been shown that T and B lymphocytes play a major role in disease development via autoantibody production and tissue infiltration, making SLE an attractive target for S1P_1_ receptor modulators [6,7].

Cenerimod is a novel, potent, selective and orally active S1P_1_ receptor modulator that has previously shown a dose-dependent reduction of circulating lymphocyte count and disease parameters in an SLE mouse model, as well as a dose-dependent reduction of lymphocyte count in patients with SLE [8,9,10]. Currently, cenerimod is being investigated in a Phase IIb study ‘CARE’ in patients with SLE (NCT03742037).

Although the effective reduction of peripheral T and B lymphocytes by cenerimod and other S1P_1_ receptor modulators has been well characterized [4,9,10,11], the direct S1P_1_ receptor-mediated chemotaxis of lymphocytes towards S1P and the inhibitory effect of S1P_1_ receptor modulators on this process has only been partially described, especially for primary human cells.

In this study, we characterized in vitro S1P_1_ receptor expression and internalization dynamics of primary human T and B lymphocytes of healthy donors in different activation states. In addition, S1P-mediated chemotaxis was analyzed using a novel real-time migration assay with subsequent characterization of the effects of cenerimod in this experimental setup. As GCs are frequently used in the treatment of patients with autoimmune disorders including SLE, the potential influence of GCs on S1P_1_ receptor expression and function was additionally investigated.

## 2. Results

### 2.1. Characterization of Ligand-Mediated S1P_1_ Receptor Internalization and Reciprocal Expression of S1P_1_ Receptor and CD69 upon Activation in Primary Human T Lymphocytes

**Figure 1:**
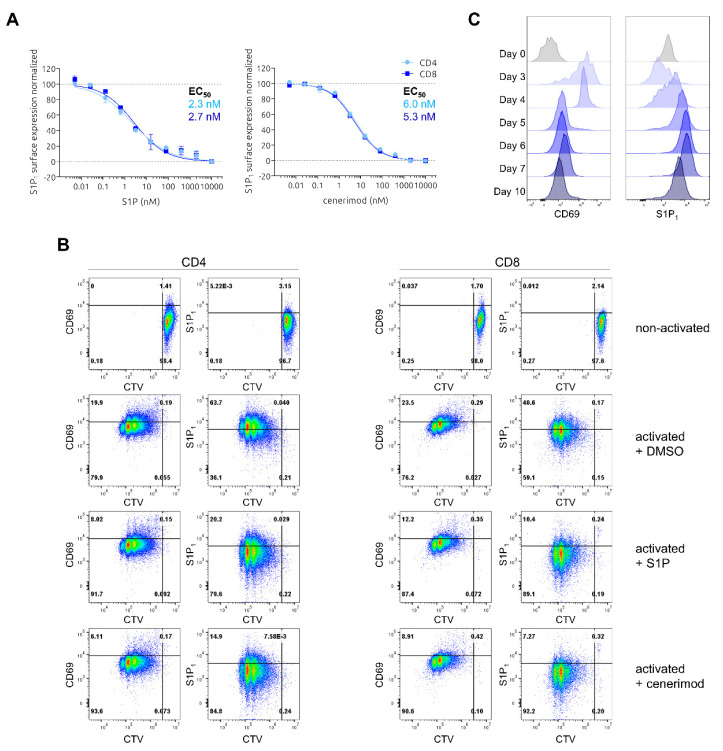
Characterization of S1P- and cenerimod-mediated S1P_1_ receptor internalization, and reciprocal S1P_1_ receptor and CD69 expression kinetics upon activation of primary human T cells. (**A**) Concentration–response curves of min/max normalized S1P_1_ receptor surface expression after 30 min ligand (S1P or cenerimod) incubation with the half maximal effective concentration (EC_50_) values given. (**B**) Detection of T cell proliferation and concurrent expression of S1P_1_ receptor and CD69. T cells were left non-activated (top) or were activated for 48 h (anti-CD3 and anti-CD28), followed by two days of proliferation, in the constant presence of S1P (0.5 μM), cenerimod (0.5 μM) or DMSO (control). (**C**) Detection of CD69 and S1P_1_ receptor expression kinetics in CD4^+^ T cells following activation by anti-CD3 and anti-CD28. CTV (CellTrace™ Violet, proliferation staining). One representative experiment from one healthy donor (*n* = 3) is shown.

To initially evaluate the impact of cenerimod on T lymphocytes, S1P_1_ receptor surface expression and internalization dynamics were characterized using flow cytometry. Primary human T cells were isolated from healthy donors and treated with different concentrations of S1P or cenerimod. Importantly, all experiments studying S1P_1_ receptor expression were performed using media lacking fetal calf serum (FCS), which is known to contain S1P, inducing unwanted immediate receptor internalization [12,13]. Both S1P and cenerimod showed an effective concentration-dependent receptor internalization, with comparable potency. Interestingly, neither S1P nor cenerimod showed any preference for either of the two CD4^+^ or CD8^+^ T cell subtypes, inducing receptor internalization at the same concentration-dependency (Figure 1A). The results obtained with cenerimod are in line with results from Strasser et al. on lymphocytes from healthy subjects and patients with SLE [9].

Lymphocyte activation is known to be accompanied by the increased expression of the activation marker CD69 on the cell surface, which has been shown to be required for lymphocyte retention in lymphoid organs in mice [14,15]. In vitro, studies using recombinant cell lines and primary mouse cells showed a direct interaction between CD69 and S1P_1_ receptor, leading to S1P_1_ receptor internalization and thereby mutual exclusion in surface expression of both proteins [16]. It is believed that this counteraction ensures a transient lymphocyte retention within the lymphoid organs to allow proper activation by antigens and subsequent coordinated egress via the S1PR-S1P gradient axes [14]. Time-dependent analysis of the phenomenon in primary human T cells, however, to our knowledge, has not been described so far. On that account, CD69 and S1P_1_ receptor surface expression kinetics upon T cell activation using samples from healthy human donors were performed and the influence of S1P or cenerimod on these processes was analyzed. Freshly isolated T cells were labeled with CellTrace™ Violet (CTV), enabling the tracing of cell divisions by dye dilution. Labeled cells were stimulated for two days with anti-CD3 and anti-CD28 antibodies in the presence of S1P, cenerimod or DMSO control. Following activation, T cells were allowed to proliferate for two consecutive days, and S1P_1_ receptor and CD69 surface expression throughout the different cell generations was tracked using flow cytometry. As seen in Figure 1B, anti-CD3 and anti-CD28 activation effectively induced T cell proliferation up to five generations compared to non-activated control. Cell activation induced high levels of CD69 surface expression that decreased progressively with increasing cell division and time. S1P_1_ receptor was almost absent on the cell surface upon initial activation and started to be re-expressed from 2nd–3rd generation in parallel with declining CD69 levels, showing the strongest expression in those cells that proliferated the most (Figure 1B,C). In this experimental setting, S1P_1_ receptor cell surface expression level was highly induced following activation compared to unstimulated T cells, where the receptor was hardly detectable. These results are similar to published data showing increased S1P_1_ receptor levels in effector T cells compared to naïve T cells in mice [17]. Cenerimod treatment, as well as S1P, strongly abolished S1P_1_ receptor surface upregulation. In addition, both cenerimod and S1P incubation, led to a slightly faster downregulation of CD69 expression with increasing cell division number (Figure 1B).

To study the apparent mutual exclusive expression of CD69 and S1P_1_ receptor kinetics over a longer post-activation time, CD4^+^ T cells were isolated and activated as described above. Subsequently, from Day 3 post activation, cell fractions were analyzed up to 10 days. As shown in Figure 1C, the induced S1P_1_ receptor up- and reciprocal CD69 downregulation magnified over the later time points, peaking at Day 6. Post Day 6, S1P_1_ receptor surface expression started to slowly decrease again, while CD69 levels continued to decrease.

Taken together, initial T cell activation led to upregulation of the activation marker CD69 and low levels of S1P_1_ receptor on the cell surface. Subsequent proliferation resulted in a strong cell surface upregulation of S1P_1_ receptor and simultaneous CD69 downregulation. This supports the model of CD69 mediating transient lymphocyte retention and subsequent egress mediated by re-expressed high levels of S1P_1_ receptor upon antigen-induced T cell activation.

### 2.2. S1P_1_ Receptor Modulator Cenerimod Mediates Effective Prevention of T Cell Migration towards S1P

S1PR modulators prevent lymphocyte egress from secondary lymphoid organs by disrupting S1PR-mediated migration along the S1P concentration gradient. In vitro, this modulation of chemotaxis was only reported using mouse T lymphocytes and mostly transwell assays systems, which restrict the investigations to single, relatively short endpoints before gradient loss [16,18,19]. Therefore, we decided to use the IncuCyte^®^ system, which allows a quantitative real-time analysis of cell migration towards an intact chemoattractant gradient by live cell imaging over several days [20]. In initial experiments, freshly isolated non-activated human T cells showed less distinct S1P-mediated chemotaxis compared to T cells in an activated state, possibly due to low S1P_1_ receptor cell surface expression (Figure S1).

**Figure 2:**
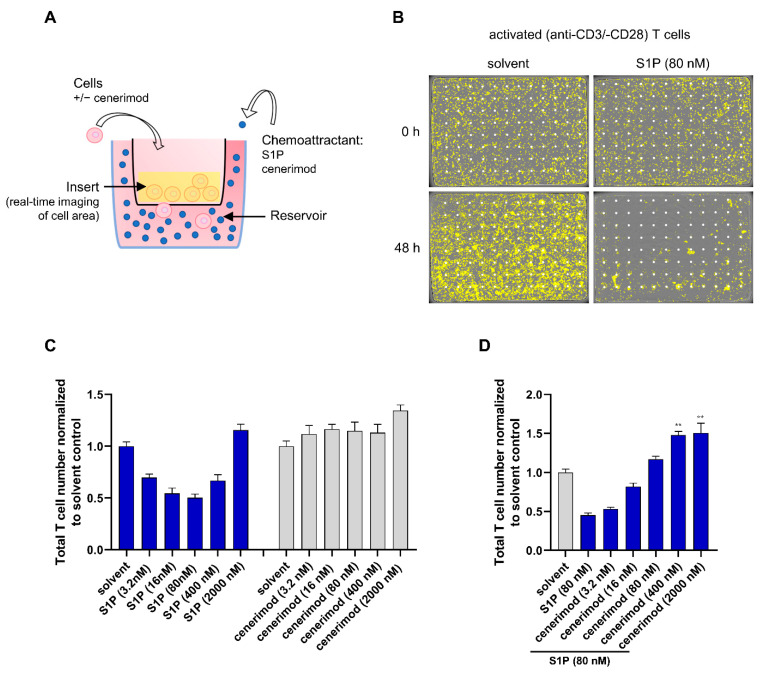
Migration of activated primary human T cells towards S1P is effectively antagonized by cenerimod. (**A**) Experimental setup of real-time migration assay towards the chemoattractant (S1P or cenerimod) with or without pretreatment of different cenerimod concentrations to test the effectiveness of migration prevention. Vertical-directed cell migration towards the chemoattractant (applied to the reservoir) was quantified by the decrease of cell counts on the top of the insert membrane over time. (**B**) Representative pictures taken of the insert surface to detect decrease of cell count due to migration. The experimental procedure was performed as described in (**A**). (**C**) Migration of activated (anti-CD3 and anti-CD28, 48 h) T cells towards different concentrations of S1P or cenerimod. (**D**) Migration of activated (anti-CD3 and anti-CD28, 48 h) T cells towards S1P (80 nM) in the presence of different cenerimod concentrations applied to cells 30 min before S1P addition as chemoattractant. (**C**/**D**) Cell migration is shown by decrease of cell count on the top of the insert membrane after 90 h of chemoattractant application, normalized to solvent control. Data are means ± SEM (technical quadruplicate). ** *p* < 0.0023 by Kruskal–Wallis nonparametric test and Dunn’s post-hoc test to compare each group to either solvent control (**C**) or S1P (80 nM only, no cenerimod) (**D**). One representative experiment from one healthy donor (*n* = 3) is shown.

Subsequent migration assays were therefore solely performed using activated T cells. The experimental setup is depicted in Figure 2A,B. Vertical-directed cell migration was quantified by the decrease of cell counts on the top of the insert membrane over time. T lymphocytes effectively migrated towards S1P in a concentration-dependent manner, with an optimal S1P concentration of 80 nM (Figure 2C and Figure S2A). Above 80 nM, directed migration decreased in a concentration-dependent manner, with the highest tested S1P concentration (2 μM) showing a slight inhibitory effect. In contrast to S1P, cenerimod as a chemoattractant induced modest to no directed migration (Figure 2C, Figures S1B and S2B).

To investigate the ability of cenerimod to prevent S1P-mediated migration of activated primary human T cells, the experimental setup was repeated using the optimal concentration of 80 nM S1P as chemoattractant. Prior to S1P application, cells were pretreated using different concentrations of cenerimod (experimental set-up depicted in Figure 2A). Cenerimod inhibited S1P-directed migration in a concentration-dependent manner, leading to complete inhibition at concentrations above 80 nM (Figure 2D and Figure S2C). 

In summary, studying real-time migration in primary human T lymphocytes showed that S1P acts as an efficient chemoattractant for this cell type. In contrast to S1P, cenerimod served as a limited chemoattractant but efficiently prevented S1P-mediated migration in a concentration-dependent manner. Thus, cenerimod’s ability to act as a functional antagonist of S1P_1_-mediated chemotaxis could be confirmed.

### 2.3. Co-Treatment with Prednisolone Has No Impact on S1P-Mediated Receptor Internalization and Migration in Primary Human T Lymphocytes

In SLE and other autoimmune disorders, GCs are commonly used and represent a cornerstone of treatment. While their broad anti-inflammatory effect can provide rapid symptom relief, GCs can have various serious side effects associated with long-term use including irreversible organ damage [21,22,23]. Therefore, effective and safe steroid-sparing therapies that can help reduce toxicity from long-term use of high-dose GCs remain an unmet medical need.

**Figure 3:**
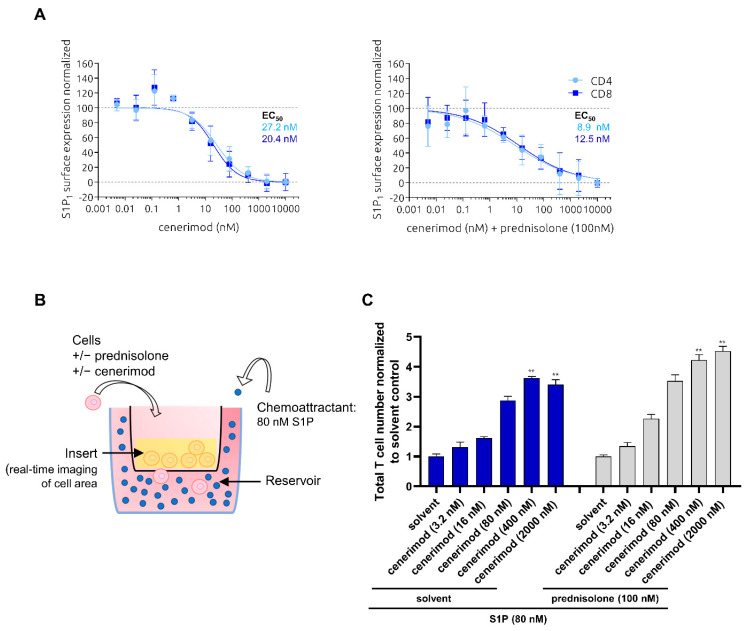
Antagonism of S1P-mediated migration and S1P_1_ receptor internalization of primary human T cells by cenerimod is not affected by prednisolone. (**A**) Concentration–response curves of min/max normalized S1P_1_ receptor surface expression after 30 min cenerimod incubation, with EC_50_ values given. As indicated, prednisolone (100 nM) or solvent control was added 30 min before cenerimod. (**B**) Experimental setup as described in Figure 2A, except that T cells were in addition treated with prednisolone or solvent control (added 30 min before cenerimod). (**C**) T cell migration towards S1P (80 nM) in the presence of different cenerimod concentrations (added 30 min before S1P). As indicated, prednisolone (100 nM) or solvent control was added 30 min before cenerimod. Cell migration is shown by decrease of cell count on the top of the insert membrane 90 h after chemoattractant application, normalized to solvent control. Data are means ± SEM (technical quadruplicate). ** *p* < 0.0048 by Kruskal–Wallis nonparametric test and Dunn’s post-hoc test to compare each group to solvent control. One representative experiment from one healthy donor (*n* = 3) is shown.

To test whether the use of GCs could have an impact on the mode of action of cenerimod, receptor internalization as well as prevention of S1P-mediated T cell migration by cenerimod was performed in the presence of prednisolone, the active metabolite of prednisone, the most commonly used GC in SLE [23]. Primary human T cells were isolated from healthy donors and treated with 100 nM prednisolone, a concentration within the therapeutic range detected in the plasma of treated patients [24]. Following prednisolone pre-incubation of 30 min, different cenerimod concentrations were applied and receptor internalization was analyzed by flow cytometry. In line with the results shown in Figure 1A, cenerimod led to an equally effective concentration-dependent S1P_1_ receptor internalization in both CD4^+^ and CD8^+^ T cell subtypes. Importantly, the presence of prednisolone did not impair cenerimod-induced receptor internalization (Figure 3A). Similar results were observed after a longer incubation time of 24 h with prednisolone (Figure S3A).

To test the potential impact of prednisolone on cenerimod’s ability to prevent T lymphocyte migration towards S1P, the same experimental setup described in Figure 2 was used. Activated T cells were pretreated with prednisolone (100 nM) or solvent control, prior to cenerimod addition at different concentrations. Following cenerimod pre-incubation, S1P (80 nM) was added as the chemoattractant (experimental set-up depicted Figure 3B).

T lymphocyte migration towards S1P was inhibited by cenerimod in a concentration-dependent manner. The presence of prednisolone showed no impact on the ability of cenerimod to inhibit T cell migration at any concentration tested over a period of 90 h (Figure 3C and Figure S3B,C). These data suggest that concomitant GC treatment has no impact on the ability of cenerimod to inhibit S1P-mediated migration of human T lymphocytes.

### 2.4. Characterization of Ligand-Mediated S1P_1_ Receptor Internalization and Reciprocal Expression of S1P_1_ Receptor and CD69 upon Activation in Primary Human B Lymphocytes

In addition to T lymphocytes, B lymphocytes represent the second group of adaptive immune cells playing a crucial role in the pathology of SLE. As observed with T cells, S1P_1_ receptor modulators impact B cells in an analogous way; preventing egress from the lymphoid organs and thereby reducing the numbers of circulating blood and autoantibody-releasing B cells [9,10].

**Figure 4:**
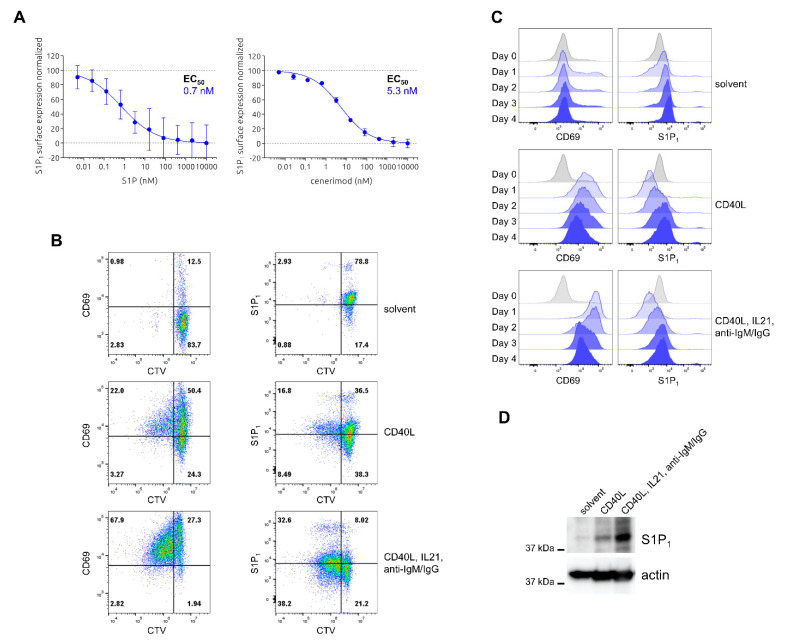
Characterization of S1P- and cenerimod-mediated S1P_1_ receptor internalization, and S1P_1_ receptor and CD69 expression kinetics upon cell activation in primary human B cells. (**A**) Concentration–response curves of min/max normalized S1P_1_ receptor surface expression after 30 min ligand (S1P or cenerimod) incubation, with EC_50_ values given. (**B**) Detection of B cell proliferation and concurrent expression of S1P_1_ receptor and CD69 after four days of B cell activation by CD40L (2 μg/mL) alone or combined activation using CD40L (2 μg/mL), IL-21 (20 ng/mL) and anti-IgM/IgG (10 μg/mL) compared to non-activated control (solvent). (**C**) Expression kinetics of CD69 and S1P_1_ receptor on B cells from Day 0 to Day 4 post activation treatment as described in (**B**). (**D**) Total S1P_1_ receptor expression level after four days of B cell activation compared to non-activated control with treatments as described in (**B**) analyzed by Western Blot. CTV (CellTrace™ Violet, proliferation staining). One representative experiment from one healthy donor (*n* = 3) is shown.

Similarly to the T lymphocyte characterization, S1P_1_ receptor surface expression and internalization dynamics by S1P and cenerimod were characterized using flow cytometry analysis of freshly isolated primary human B cells. S1P and cenerimod treatment led to a concentration-dependent receptor internalization on B cells (Figure 4A). This was similar to receptor internalization observed on T cells (Figure 1A) and in line with results reported by Strasser et al. [9].

As previously reported for T cells, the activation of B cells leads to an upregulation of CD69 and the reciprocal downregulation of the S1P_1_ receptor on the cell surface, leading to B cell retention within lymphoid organs in mice [15]. However, time-dependent analysis of this phenomenon in primary human cells is not available. S1P_1_ receptor and CD69 surface-expression kinetics were performed using freshly isolated B cells from healthy donors activated by using various stimuli or left untreated. After CTV staining for cell division tracing, B cells stimulated with CD40L alone or CD40L in combination with IL-21 and anti-IgM/IgG (triple activation) were compared to a non-activated control. From Day 0 to Day 4 post activation, flow cytometry was used to analyze S1P_1_ receptor and CD69 surface expression throughout the different cell generations and time. As indicated in Figure 4B, triple activation led to robust induction of proliferation up to three generations at Day 4 versus control. Treatment with CD40L alone led to modest proliferation induction at Day 4 compared to non-activated control cells.

Similar to the observations in T cells, B cell activation led to a rapid upregulation of CD69 at Day 1 (generation 0) induced by both CD40L alone and more pronounced with triple activation. CD69 levels slowly decreased over time, but in contrast to observations in T cells, surface expression levels stayed relatively high throughout the entire experiment and remained higher than levels prior to activation. S1P_1_ receptor surface expression was immediately downregulated on Day 1 post activation, with a more pronounced effect by triple activation compared to CD40L alone (Figure 4B,C). A modest S1P_1_ receptor re-expression was observed over time, seemingly independent of CD69 presence on the cell surface. Non-activated B cells kept the highest S1P_1_ receptor surface expression compared to activated B cells even at the last post-activation day analyzed (Figure 4B,C), in contrast to observations made in T cells. Despite the lack of enhanced S1P_1_ cell surface expression detected in activated B cells, B cells at Day 4 showed a marked increase of total S1P_1_ receptor expression upon activation (CD40L alone < triple activation) when analyzing the total protein expression levels in cell lysates by Western Blot (Figure 4D). As seen for T cells, concomitant treatment of cenerimod and S1P reduced S1P_1_ receptor surface upregulation upon activation, accompanied by faster downregulation of CD69 expression with increasing cell division number (Figure S4).

In summary, B cell activation led to the expected upregulation of the activation marker CD69 and downregulation S1P_1_ receptor, as well as induction of proliferation by the strongest activation treatment. However, in contrast to the observations made in T lymphocytes, S1P_1_ receptor re-expression was induced even with clearly detectable CD69 surface expression. Furthermore, total S1P_1_ receptor expression, in contrast to surface expression, was clearly induced by B cell activation. 

### 2.5. S1P_1_ Receptor Modulator Cenerimod Effectively Prevents B Cell Migration towards S1P

In vitro data directly showing S1P-mediated B cell migration and its regulation by S1P_1_ receptor modulators using long-term, real-time assays in primary human cells have not been reported [25,26]. Therefore, using the previously described system, B cell migration and the mode of action of cenerimod was analyzed using freshly isolated human B cells. Based on the results shown in Figure 4, it was unclear whether non-activated or activated B cells were more prone to migrate in our experimental system. Therefore, cells in both states were tested for migration towards S1P and cenerimod. As described above, vertical-directed cell migration was quantified over time.

**Figure 5:**
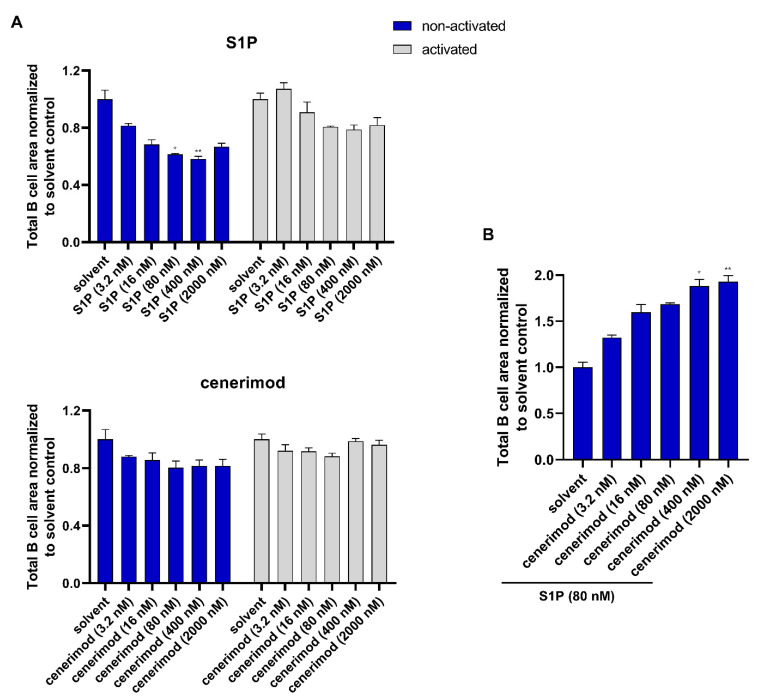
Migration of primary human B cells towards S1P is effectively antagonized by cenerimod. (**A**) B cell migration towards different concentrations of S1P and cenerimod. B lymphocytes were activated with CD40L (2 μg/mL), IL-21 (20 ng/mL) and anti-IgM/IgG (10 μg/mL) for 48 h or left untreated. (**B**) Non-activated B cell migration towards S1P (80 nM) in the presence of different cenerimod concentrations applied to cells 30 min before the addition of S1P as chemoattractant. (**A**/**B**) Cell migration is shown by decrease of cell area on the top of the insert membrane after 30 h of chemoattractant application, normalized to solvent control. Data are means ± SEM (technical triplicate). * *p* < 0.0184, ** *p* < 0.0066 by Kruskal–Wallis nonparametric test and Dunn’s post-hoc test to compare each group to solvent control. One representative experiment from one healthy donor (*n* = 3) is shown.

Non-activated B cells effectively migrated towards S1P in a concentration-dependent manner, with the same optimal concentration range (80 nM) as observed for activated T cells. At concentrations of S1P above 400 nM, directed migration decreased concentration-dependently, resulting in a bell-shaped migration curve similar to that observed with T cells. Activated B cells migrated poorly towards S1P, but within the same concentration range as non-activated cells (Figure 5A showing 30 h time point and Figure S5A,B). Cenerimod as a chemoattractant did not lead to migration in non-activated nor activated B cells at any concentration tested (Figure 5A and Figure S5A,B). 

To study the functional antagonism of cenerimod on S1P-mediated B cell migration, S1P at an optimal concentration of 80 nM was applied as a chemoattractant to non-activated B cells, which showed a stronger migratory behavior. Before S1P application, cells were pretreated with different cenerimod concentrations. As observed for T lymphocytes, cenerimod effectively inhibited S1P-mediated migration in a concentration-dependent manner (Figure 5B and Figure S5C).

Taken together, S1P acts as an efficient chemoattractant in primary human B lymphocytes with an optimal concentration response between 80 nM–400 nM. Interestingly in contrast to the observations made in T lymphocytes, more B cells migrated in a non-activated state compared to after cell activation. Contrary to S1P, cenerimod did not act as a chemoattractant, but efficiently prevented S1P-mediated migration in a concentration-dependent manner.

### 2.6. Co-Treatment with Prednisolone Has No Impact on the Ability of Cenerimod to Inhibit S1P-Mediated Migration in Primary Human B Lymphocytes

**Figure 6:**
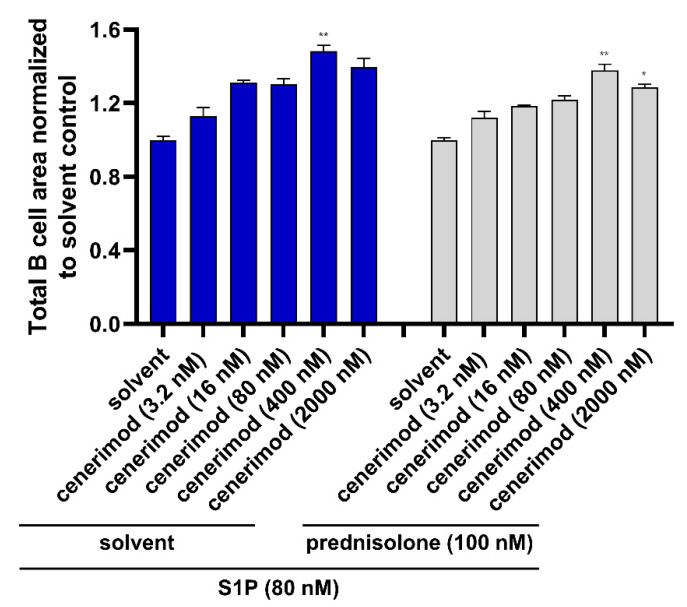
Antagonism of S1P-mediated migration of primary human B cells by cenerimod is not affected by prednisolone. Non-activated B cell migration towards S1P (80 nM) in the presence of different cenerimod concentrations applied to cells 30 min before the addition of S1P as chemoattractant. As indicated prednisolone (100 nM) or solvent control was added 30 min before cenerimod. Cell migration is shown by decrease of cell area on the top of the insert membrane after 30 h of chemoattractant application, normalized to solvent control. Data are means ± SEM (technical triplicate). * *p* < 0.0419, ** *p* < 0.0029 by Kruskal–Wallis nonparametric test and Dunn’s post-hoc test to compare each group to solvent control. One representative experiment from one healthy donor (*n* = 3) is shown.

To test whether the use of GCs could have an impact on the mode of action of cenerimod in B cells, prevention of S1P-mediated primary B cell migration was performed in the presence of prednisolone. B lymphocytes were pretreated with prednisolone or solvent control before cenerimod was added at different concentrations. After cenerimod pre-incubation, S1P (80 nM) was added as the chemoattractant. The results in Figure 6 and Figure S6 demonstrate the effective concentration-dependent inhibition of S1P-mediated B cell migration by cenerimod. In this setting, the presence of prednisolone showed no impact on cenerimod’s mode of action at any tested concentration. 

## 3. Discussion

S1P_1_ receptor modulators have shown their therapeutic potential in autoimmune disorders such as RMS and UC [2]. These examples demonstrate that S1P_1_ receptor modulators can be efficacious therapies in certain autoimmune diseases by acting as functional antagonists on the S1P_1_ receptor, resulting in the trapping of autoreactive lymphocytes within the secondary lymphoid organs. Therefore, this mechanism of action may be applicable for the treatment of other autoimmune-related diseases with a high unmet medical need. This concept of interfering with the trafficking of lymphocytes has particular relevance for the treatment of SLE, a complex autoimmune disease with substantial involvement of autoreactive T and B cells in disease development [6,7].

Cenerimod, a novel, selective S1P_1_ receptor modulator has shown promising results in a relevant SLE mouse model as well as in a proof-of-concept study in patients with SLE, suggesting that cenerimod has the potential to treat patients with SLE [8,9,10]. In both studies, a dose-dependent reduction of circulating lymphocytes by cenerimod treatment was observed that was also accompanied by a decrease of disease activity.

In this study, the molecular mechanism of cenerimod acting on primary human T and B lymphocytes in the context of S1P_1_ receptor expression and S1P-mediated cell migration was studied in detail.

A hallmark of S1P_1_ receptor modulators is the effective induction of receptor internalization and desensitization after receptor engagement. Therefore, we first characterized S1P_1_ receptor expression on human T and B cells upon cenerimod treatment. For both cell types, cenerimod induced concentration-dependent internalization of the receptor similar to the natural ligand (Figure 1A and Figure 4A), confirming previously reported data and verifying the proposed mode of action of cenerimod as an S1P_1_ receptor modulator [9].

The dynamics of receptor internalization and desensitization upon activation and subsequent re-expression leading to the restoration of S1P_1_ receptor sensitivity is key for the constant lymphocyte circulation between secondary lymphoid organs and the blood [2]. Gaining more insight into the underlying kinetics of receptor expression in this context and in relation to S1P_1_ receptor modulation is an important aspect to improve our understanding of the effects of cenerimod on lymphoid cell trafficking.

Analyzing S1P_1_ receptor expression in human T and B lymphocytes post cell activation clearly showed that in both cell types stimulation led to a quick loss of detectable S1P_1_ receptor on the cell surface. This loss in S1P_1_ receptor surface expression was paralleled by subsequent cell proliferation and slow re-expression of the receptor on the cell surface over time. T cells in a non-activated state showed relatively low S1P_1_ receptor surface expression levels; however, these levels strongly increased four days after T cells activation (Figure 1B,C). In comparison to T cells, B cells in a non-activated versus activated state possessed a higher surface expression of the S1P_1_ receptor, which confirmed results from Strasser et al. [9]. Intriguingly, in contrast to what was seen in T cells, the surface expression levels of non-activated B cells were not reached by re-expression even four days post activation (Figure 4B,C), whereas total cellular S1P_1_ receptor expression was seen to be highly increased upon activation during that time period (Figure 4D).

S1P_1_ receptor expression upon lymphocyte activation is strongly associated with expression and interaction of the activation marker CD69. In fact, the upregulation of CD69 was shown to be required for mediating S1P_1_ receptor downregulation and thereby lymphocyte retention [14,15,16]. In the case of T lymphocytes, our data confirmed this mutually exclusive expression of CD69 and S1P_1_ receptor upon activation over time. For B cells, CD69 surface expression slowly declined following the initial strong upregulation after activation, but total expression remained elevated even with S1P_1_ receptor re-expression on the cell surface (Figure 4B,C).

As most studies investigating S1P_1_ receptor and CD69 regulation were performed in mouse models or cell lines, it appears that in the case of primary human B cells, the surface expression of CD69 and S1P_1_ receptor is not as strictly mutually exclusive as confirmed on human T cells. Further studies are required to identify potential underlying differences in signaling events between T and B lymphocytes, such as CD69-S1P_1_ receptor interaction behavior.

By testing the effect of cenerimod on CD69 and S1P_1_ receptor expression kinetics post activation in T and B cells, we could show that cenerimod leads not only to an effective downregulation of the S1P_1_ receptor but is also able to restrict S1P_1_ receptor re-expression after cell activation (Figure 1B and Figure S4). These data demonstrate that cenerimod efficiently mediates a consistent downregulation of the S1P_1_ receptor in lymphocytes at different activation states in vitro, which in vivo may translate into the prevention of the migrational egress of autoreactive, activated lymphocytes from secondary lymphoid organs into circulation.

Published studies analyzing S1P-mediated lymphocyte migration, are mostly limited to mouse lymphocytes or cell lines, using traditional transwell assays and are hence single, short endpoint experimental setups [16,18,19]. To generate more clinically relevant data, we took advantage of a novel real-time migration assay that allows monitoring S1P_1_-dependent migration of primary human T and B lymphocytes over a prolonged period of time. Both lymphocyte subtypes showed a clear concentration-dependent migration towards S1P over the tested time course up to 90 h. Interestingly, both T and B cells showed optimal migration at the same S1P concentration of 80 nM. Concentrations above 80 nM led to a concentration-dependent decline of cell migration with the highest concentration showing a slight inhibitory effect (Figure 2C and Figure 5A). This phenomenon is well described for chemokines, where high concentrations of chemoattractants are limiting chemotaxis resulting in a typically bell-shaped concentration–response curve [27].

We also tested lymphocyte migration in the context of different cell activation states. Consistent with the previous characterization of S1P_1_ receptor surface expression kinetics, T cells expressing the highest S1P_1_ receptor levels two days post activation migrated efficiently towards the S1P gradient. In contrast, non-activated T cells showed only minor directed migration towards S1P suggesting that basal receptor expression in those cells was limiting migration in this experimental setup. In B cells, S1P_1_ surface receptor levels in the non-activated state were higher compared to any post activation state, most likely leading to non-activated B lymphocytes migrating more effectively than B cells activated by triple activation (a co-treatment of CD40L, IL-21 and anti-IgM/IgG). With this set of data, we could show that cell activation can increase S1P-mediated lymphocyte migration as seen in the case of T cells. However, the timeframe after activation defining the S1P_1_ receptor surface expression status is crucial. As such, shortly after activation, where receptor re-expression has not yet occurred, T cell migration is diminished as reported by Graeler et al. [28]. At later timepoints however effector T cells with enhanced S1P_1_ receptor surface expression show a stronger migrational behavior [17]. For B cells, unlike for T cells, S1P_1_ receptor surface re-expression levels following activation did not exceed S1P_1_ receptor levels on the surface of non-activated cells, most likely explaining their reduced migrational activity. However, it cannot be excluded that receptor cell surface re-expression dynamics in B cells take longer than four days post activation—the experimental timeframe could not be extended due to cell viability in vitro, which was lower for activated B cells compared to activated T cells in the tested conditions. Nevertheless, as shown by Western blotting, a strong induction of total S1P_1_ receptor protein production was detected in activated B cells at Day 4, which did not result in increased cell surface expression. The biological significance of this observation would need further investigation.

As stated previously, a key rationale of the application of S1P_1_ receptor modulators in autoimmune diseases is to prevent the circulation of autoreactive lymphocytes. Importantly, cenerimod was able to efficiently prevent S1P-mediated migration for both T and B cells in a concentration-dependent manner. With cenerimod acting as a selective S1P_1_ receptor modulator, this study confirms the S1P_1_ receptor as the responsible S1P receptor in lymphocyte chemotaxis towards S1P in this context [18,19]. These in vitro data using primary human cells complement on a molecular level in vivo data reported by Strasser et al. and Hermann et al., showing significant reduction of peripheral T and B lymphocytes by cenerimod in mice and human SLE patients. Importantly, concomitant treatment with prednisolone had no impact on the mode of action of cenerimod preventing S1P-mediated migration.

In summary, we were able to further elucidate the underlying molecular principles of human T and B cell migration towards the chemoattractant S1P and confirm the effectiveness and mode of action of the novel S1P_1_ receptor modulator cenerimod in this biological context, taking one more step in tackling the treatment of autoimmune diseases.

## 4. Materials and Methods

### 4.1. Receptor Internalization Assay

Blood from healthy donors was collected in EDTA blood collection tubes, and T and B lymphocytes were isolated using MACSxpress Cell Isolation Kits (Miltenyi, pan T Cell #130-098-193, B Cell #130-098-190). The choice of isolation method is crucial for studying S1P_1_ receptor in vitro as in-house data have shown that lymphocytes isolated with MACSxpress preserve S1P_1_ receptor surface expression in contrast to density gradient isolation. Cells were taken up in OpTmizer T Cell Expansion Medium (Gibco, A1048501) containing the corresponding supplement, 2 mM L-glutamine and penicillin–streptomycin (P/S). Importantly, all experiments studying S1P_1_ receptor expression were performed using this special OpTmizer medium, lacking FCS, which is known to potentially contain S1PR ligands, inducing unwanted immediate receptor internalization. Therefore, cells were cultured overnight to allow S1P_1_ receptor surface re-expression. Cenerimod and S1P (Tocris, #1370) were added in serial dilution to cells and incubated at 37 °C for 30 min/24 h. For testing the effect of GCs, prednisolone (100 nM) was preincubated before ligand addition for another 30 min at 37 °C. Then cells were washed once and subsequently stained using the following antibodies: CD3_BD Horizon™ V450_ (BD Bioscience, 560365), CD4_Brilliant Violet 785™_ (BioLegend, 317442), CD19_Brilliant Violet 510™_ (BioLegend, 302242), S1P_1_ receptor_eFluor__660_ (eBioscience, 50-3639-42). Every condition was performed with technical replicates. One representative experiment (*n* = 3) from one healthy donor is shown per figure. Samples were measured using NovoCyte/NovoCyte Quanteon (ACEA Biosciences Inc. (Santa Clara, CA, USA)) and subsequently analyzed by Flowjo software (Becton Dickinson) and Prism (graphpad).

### 4.2. CTV Proliferation Assay

B and T lymphocytes were isolated and taken up in OpTmizer T Cell Expansion Medium as described above. Cells were then labeled with CellTrace™ Violet (CTV) cell proliferation Kit according to the manufacturer’s protocol (LifeSciences, C34557). Cells were left untreated or activated as follows: T cells were activated by adding human T activator CD3/CD28 Dynabeads^TM^ according to manufacturer’s protocol (Gibco, 11132D), which were removed after 48 h/72 h, and replaced by fresh media, containing IL-2 (30 U/mL (Miltenyi, 130-097-743)) to promote T cell survival and proliferation. B cells were either activated by CD40L (2 μg/mL (BioLegend, BLG-591706)) alone or in combination with IL-21 (20ng/mL (Miltenyi, 130-094-563)) and F(ab’)2-Goat anti-Human IgG, IgM (H+L) Secondary Antibody (10 μg/mL (eBioscience, 16-5099-85)). For co-treatment with S1P or cenerimod, application occurred right after CTV labeling and S1P or cenerimod addition was refreshed after 48 h. Every 24 h, one fraction of cells was washed and stained with the following antibodies for flow cytometry analysis: CD69_Alexa Fluor 488_ (BioLegend, 310916)/CD69_PE-Vio770_ (Miltenyi, 130-113-525), S1P_1_ receptor_eFluor__660_ (eBioscience, 50-3639-42), CD19_PE_ (BioLegend, 302208), CD4_PE_ (BioLegend, 300508). Every condition was performed with technical replicates. One representative experiment from one healthy donor (*n* = 3) is shown per figure. Samples were measured using NovoCyte/NovoCyte Quanteon (ACEA Biosciences Inc.) and subsequently analyzed by Flowjo software (Becton Dickinson) and prism (graphpad). 

### 4.3. Real-Time Migration Assay (IncuCyte™)

T and B lymphocytes were isolated and taken up in OpTmizer T Cell Expansion Medium as described above. Cells were left untreated or activated as described in Section 4.2 following 48 h of incubation at 37 °C. Then cells were counted and seeded at a density of 5000 cells/well on precoated inserts of IncuCyte™ ClearView 96-Well Chemotaxis Plates (Essen BioScience, 9600-15). Inserts of those plates contain a limited number of 8 μM pores that allow to maintain a chemoattractant gradient for up to three days. Coating was performed by incubating in the first step with recombinant protein G (20 μg/mL (Invitrogen, 101200)) at 37 °C for 1 h, followed by an incubation with human ICAM-1/CD54 protein (15 µg/mL (Sino Biological, 10346-H03H)) for 2 h at 37 °C. After cell seeding, prednisolone (100 nM), if applied, was added to the insert for a 30 min incubation at room temperature (RT). In the next step, cenerimod was added in serial dilutions to the insert for an additional 30 min incubation (RT), before S1P or cenerimod as chemoattractants were finally added to the reservoir of the 96-well plate. Every condition was performed with technical replication. One representative experiment from one healthy donor (*n* = 3) is shown per figure. Cell migration was measured by taking whole-well images in a 2 h or 3 h time-lapse, using the IncuCyte^®^ ZOOM/SX5 (Sartorius), allowing vertical-directed cell migration to be quantified by the decrease of cell count on the top of the insert membrane over time. Data were analyzed using IncuCyte^®^ ZOOM 2016B software (Sartorius) and prism (graphpad).

### 4.4. Western Blot Analysis

B lymphocytes were isolated and activated as described above. Cells were washed with PBS before the addition of lysis buffer (TBS containing 0.5% NP40, 1% Triton X-100, 1 mM EDTA, 1× protease inhibitor cocktail complete (Roche, 11836170001), phosphatase inhibitor cocktail (life technologies, A32957) followed incubation on ice (10 min). Lysates were cleared by centrifugation and taken up in NuPage LDS buffer (Invitrogen, #NP-0007) together with 20 mM DTT. Proteins were separated on 4–20% Criterion™ TGX Stain-Free™ Protein Gel (Biorad, 5678095), using corresponding running buffer Tris/Glycine/SDS (Biorad, 1610732). Subsequent immunoblotting on PVDF membranes (Biorad, 1704157) was performed using the Biorad Trans-Blot^®^ Turbo™ Transfer System. Proteins of interest were detected using the following antibodies: anti-S1P_1_ receptor (Biorad, MCA5960GA) anti-Actin (cell signaling, #4970), diluted in 3% milk/PBS–Tween (0.1%). One representative experiment from one healthy donor (*n* = 3) is shown per figure.

### 4.5. Statistical Analysis

All data are expressed as mean of technical replicates ± standard error of the mean (SEM). Each experiment was performed *n* = 3 using material from different human healthy donors. Statistical analysis was performed using Prism version 8.1.1 (GraphPad software, San Diego, CA, USA). The Kruskal–Wallis nonparametric test was applied following post-hoc analysis using Dunn’s test to compare each group to solvent control. *p*-values were adjusted for multiple comparisons. Differences were considered significant at *p*-values < 0.05.

## Data Availability

Data are contained and available within this manuscript.

## Supplementary Materials

The following are available online at https://www.mdpi.com/article/10.3390/ijms23031191/s1.
