# Supplementary material for: In Vitro Characterization of Sphingosine 1-Phosphate Receptor 1 (S1P1) Expression and Mediated Migration of Primary Human T and B Cells in the Context of Cenerimod, a Novel, Selective S1P1 Receptor Modulator
Source: Int J Mol Sci. 2022 Jan 21;23(3):1191. doi: 10.3390/ijms23031191 (PMC8835580; doi:10.3390/ijms23031191)
Supplement: Supplementary file 1 [file ijms-23-01191-s001.zip › ijms-1508801-supplementary.pdf]

Supplementary Information

In vitro Characterization of Sphingosine 1-Phosphate Receptor 1 ($S1P_1$) Expression and Mediated Migration of Primary Human T and B Cells in the Context of Cenerimod, a Novel, Selective S1P1 Receptor Modulator

Lisa Schlicher, Paulina Kulig, Audrey von Münchow, Mark J Murphy and Marcel P Keller

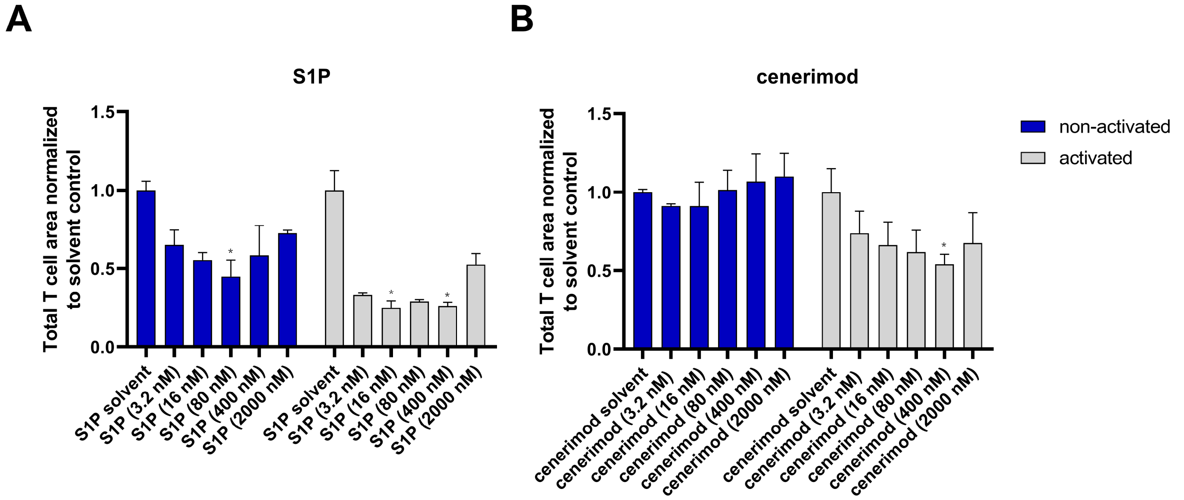

Figure S1. Comparison of migration of non-activated versus activated primary human T cells towards S1P and cenerimod. T cells were activated (anti-CD3 and anti-CD28, 48 h) or left untreated. Cell migration towards different concentrations of (**A**) S1P or (**B**) cenerimod is shown by decrease of cell area on the top of the insert membrane after 90 h of ligand application, normalized to solvent control. Data are means ± SEM (technical triplicate). * $p < 0.0372$ by Kruskal-Wallis nonparametric test and Dunn's post-hoc test to compare each group to solvent control. One representative experiment from one healthy donor (n=3) is shown.

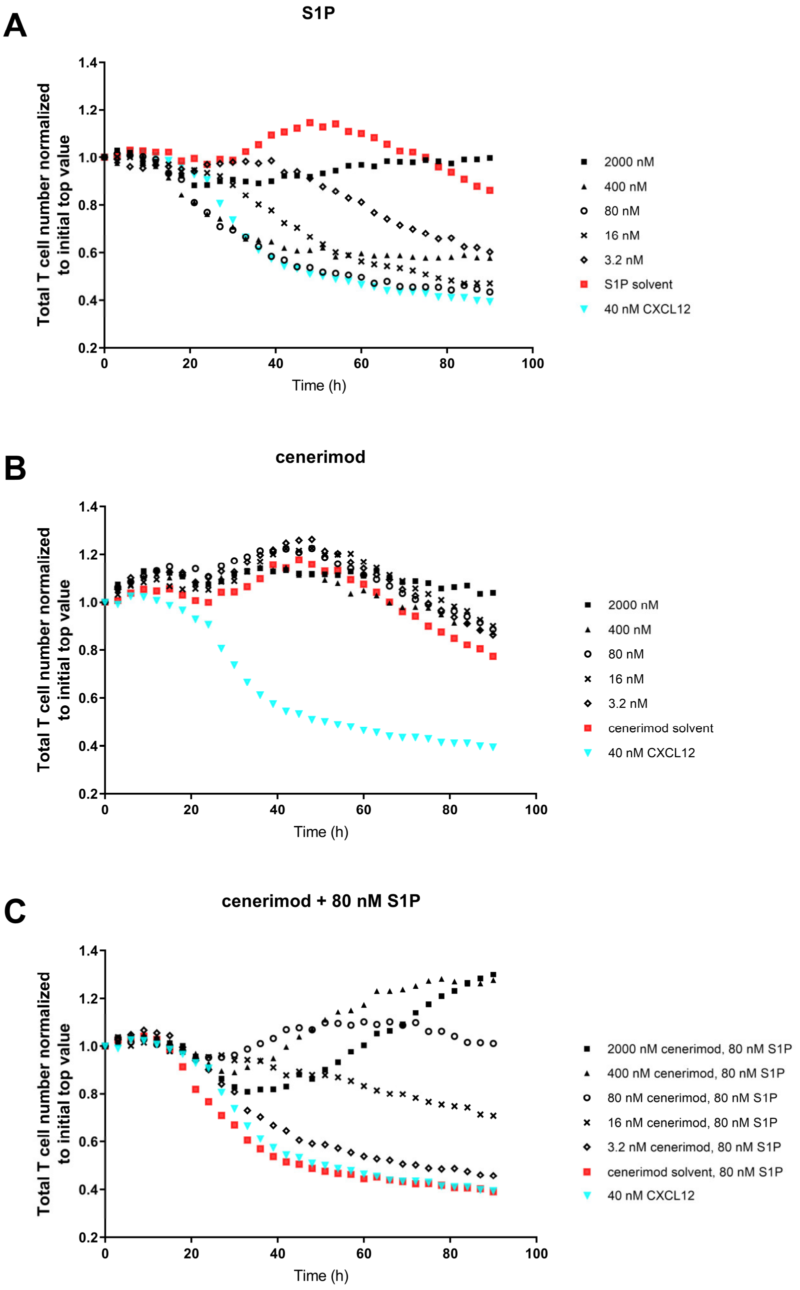

Figure S2. Migration kinetics of activated primary human T cells towards S1P/ cenerimod and migration kinetic towards S1P effectively antagonized by cenerimod. (**A**) Migration of activated (anti-CD3 and anti-CD28, 48 h) T cells towards different concentrations of S1P. (**B**) Migration of activated (anti-CD3 and anti-CD28, 48 h) T cells towards different concentrations of cenerimod. (**C**) Migration of activated (anti-CD3 and anti-CD28, 48 h) T cells towards S1P (80 nM) in the presence of different cenerimod concentrations applied to cells 30 minutes before S1P addition as chemoattractant. CXCL12 serving as positive migration control. Cell migration is shown by decrease of cell count on the top of the insert membrane normalized to initial top cell count value. Pictures for cell count taken every 2 h up to 90 h.

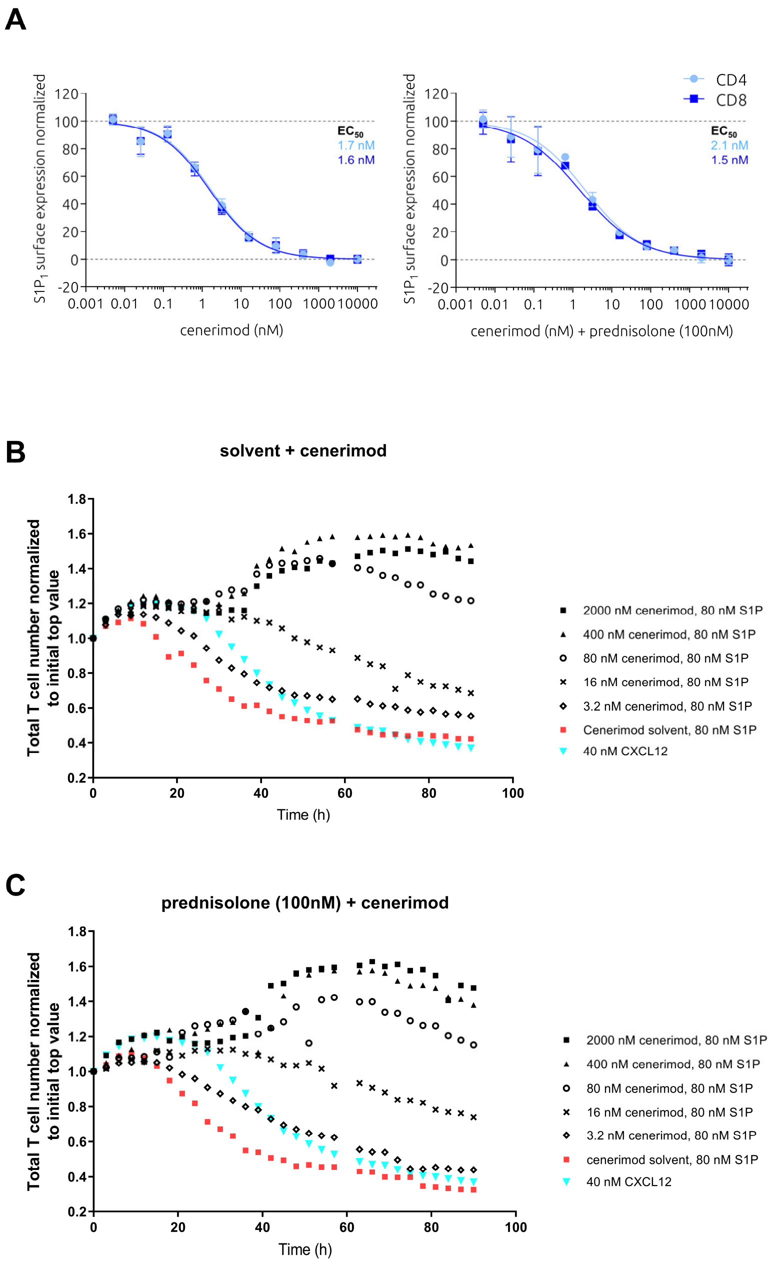

Figure S3. Antagonism of S1P-mediated migration kinetic and $S1P_1$ receptor internalization of primary human T cells by cenerimod is not affected by prednisolone. (**A**) Concentration-response curves of min/max normalized $S1P_1$ receptor surface expression after 24 h cenerimod incubation, with EC_{50} values given. As indicated prednisolone (100 nM) or solvent control was added 30 minutes before cenerimod. (B,C) Migration of activated (anti-CD3 and anti-CD28, 48 h) T cells towards S1P (80 nM) prevented by addition of cenerimod at different concentrations (added 30 min before S1P). As indicated prednisolone (100 nM (C)) or solvent control (B) was added 30 minutes before cenerimod. CXCL12 serving as positive migration control. Cell migration is shown by decrease of cell count on the top of the insert membrane normalized to initial top cell count value. Pictures for cell count taken every 3 h up to 90 h.

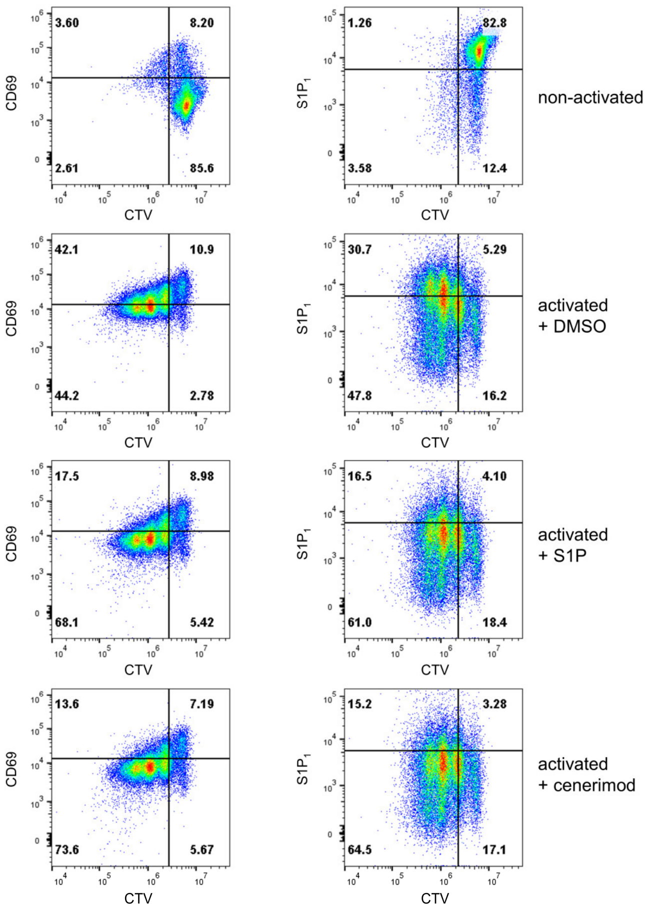

Figure S4. Characterization of S1P- and cenerimod-mediated $S1P_1$ receptor internalization, and reciprocal $S1P_1$ receptor and CD69 expression kinetics upon activation of primary human B cells. Detection of B cell proliferation and concurrent expression of $S1P_1$ receptor and CD69 after four days of B cell activation by CD40L (2 ug/mL), IL-21 (20 ng/mL) and anti-IgM/IgG (10 ug/mL) in the constant presence or absence of S1P (0.5 μM) or cenerimod (0.5 μM), compared to non-activated and DMSO control. CTV (CellTrace™ Violet, proliferation staining).

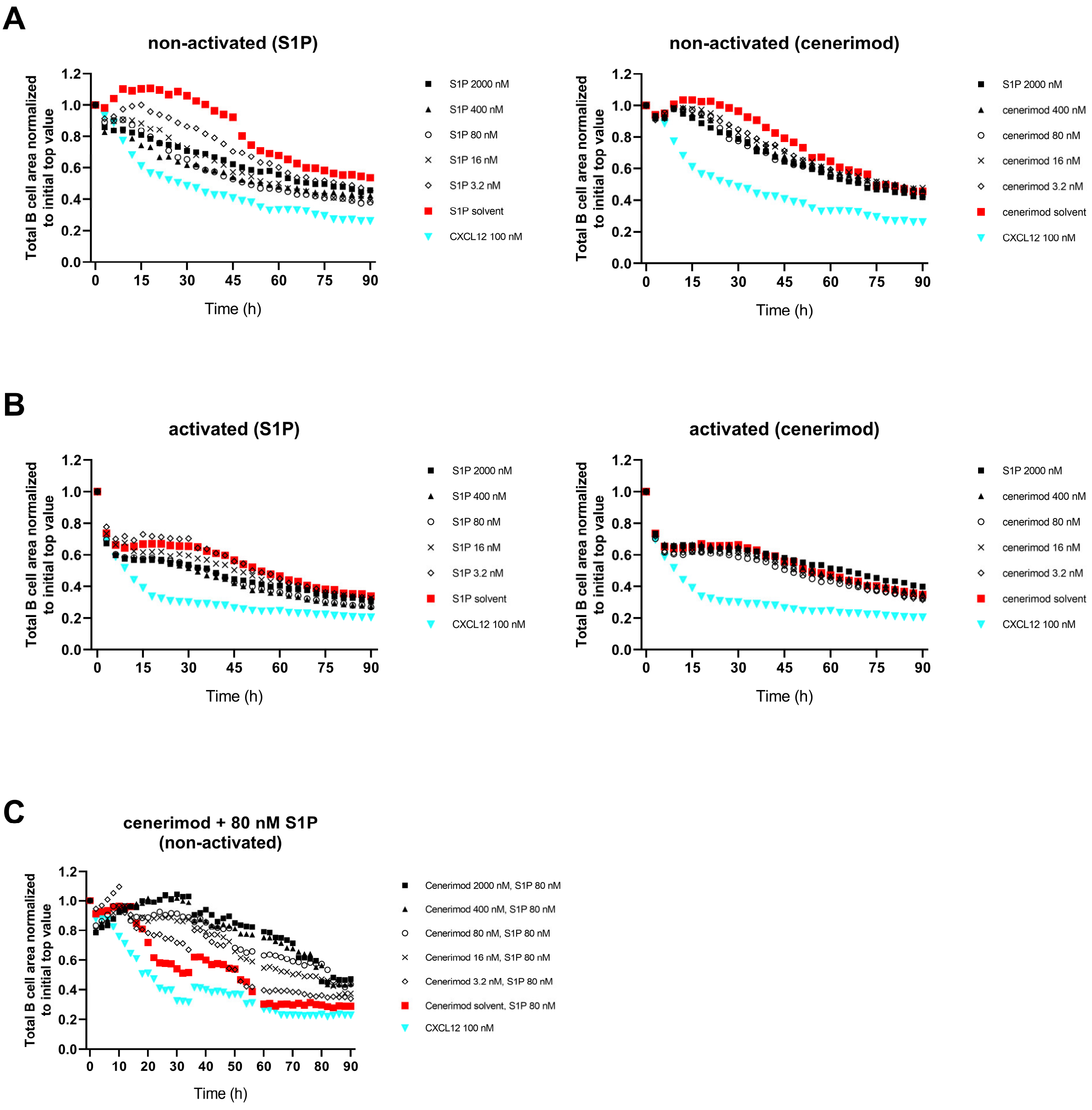

Figure S5. Migration kinetics of primary human B cells towards S1P and cenerimod, and migration kinetic towards S1P effectively antagonized by cenerimod. (**A**) Migration of non-activated B cells towards different concentrations of S1P and cenerimod. (**B**) Migration of activated B cells towards different concentrations of S1P and cenerimod. (**C**) Migration of non-activated B cells towards S1P (80 nM) in the presence of different cenerimod concentrations applied to cells 30 minutes before S1P addition as chemoattractant. CXCL12 serving as positive migration control. Cell migration is shown by decrease of cell area on the top of the insert membrane normalized to initial top cell area value. Pictures for cell area taken every 3 h (**A**,**B**) or every 2 h (**C**) up to 90 h.

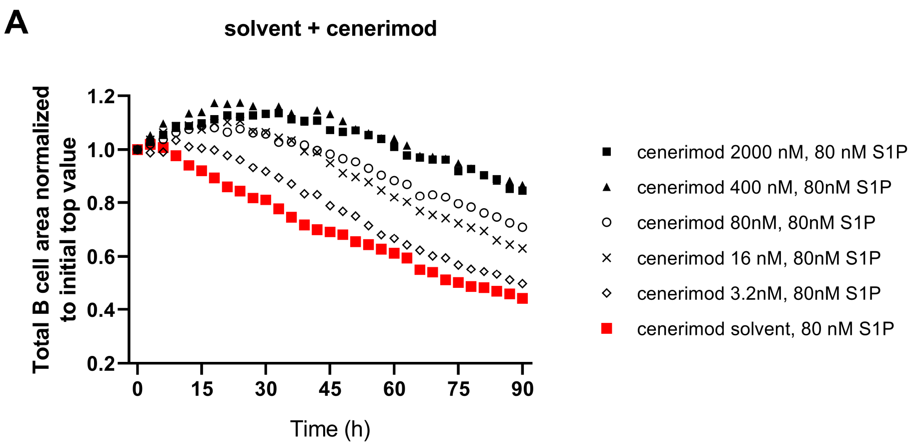

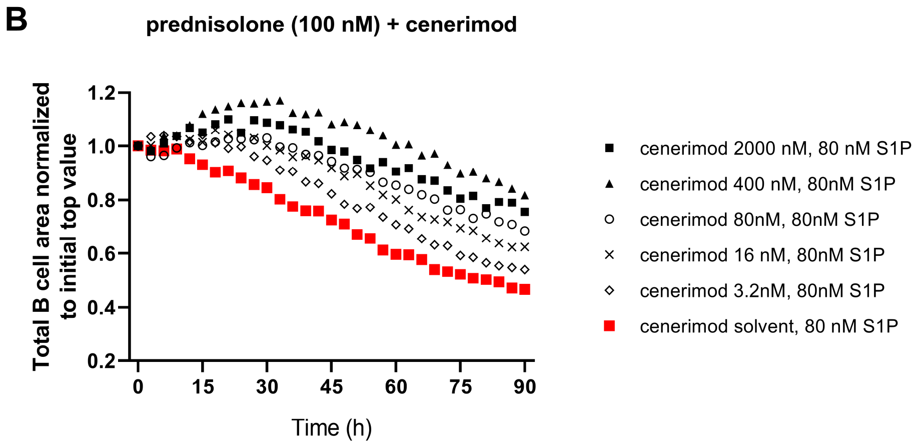

Figure S6. S1P mediated migration kinetics of non-activated primary human B cells effectively antagonized by cenerimod is unaffected by prednisolone. Migration of non-activated B cells towards S1P (80 nM), in the presence of different cenerimod concentrations applied to cells 30 minutes before S1P addition as chemoattractant. As indicated prednisolone (100 nM) or solvent control was added 30 minutes before cenerimod. Cell migration is shown by decrease of cell area on the top of the insert membrane normalized to initial top cell area value. Pictures for cell area taken every 3 h up to 90 h.
